# Multimodal Imaging in AIDS-Related Ocular Cryptococcosis

**DOI:** 10.1155/2021/8894075

**Published:** 2021-02-10

**Authors:** Maria Fernanda Flores Herrera, Nicolas Dauby, Evelyne Maillart, Agnes Libois, Alberto Papaleo, Hind El Ouardighi, Laurence Postelmans, François Willermain, Dorine Makhoul

**Affiliations:** ^1^Department of Ophthalmology, CHU Saint-Pierre, Université Libre de Bruxelles (ULB), Brussels, Belgium; ^2^Department of Infectious Diseases, CHU Brugmann, Université Libre de Bruxelles (ULB), Brussels, Belgium; ^3^Department of Infectious Diseases, CHU Saint-Pierre, Université Libre de Bruxelles (ULB), Brussels, Belgium; ^4^Department of Ophthalmology, CHU Brugmann, Université Libre de Bruxelles (ULB), Brussels, Belgium

## Abstract

**Purpose:**

To report multimodal imaging findings in two cases of AIDS-related cryptococcal chorioretinitis associated with uveitis and vasculitis.

**Methods:**

Findings on clinical examination, color fundus photography, fluorescein and indocyanine green angiographies, and optical coherence tomography. *Patients*. Both patients were diagnosed with *Cryptococcus neoformans* meningitis in the setting of untreated HIV infection with CD4+ T cell count < 100/mm^3^. Ocular manifestations occurred during the course of the antifungal therapy for meningitis.

**Results:**

In both cases, fundus showed vitritis. Fluorescein angiography allowed the characterization of vasculitis lesions, and indocyanine green angiography indicated choroidal involvement. In combination with optical coherence tomography, ICG and FA allowed the assessment of treatment response.

**Conclusion:**

These two cases reveal the potential of *C. neoformans* to infect almost all ocular structures and the critical role of multimodal imaging in baseline evaluation and in the follow-up of patients.

## 1. Introduction

Cryptococcosis is an invasive mycosis mainly caused by two species: *Cryptococcus neoformans* and *Cryptococcus gattii* that can affect both immunocompromised and immunocompetent subjects. *C. neoformans* is a major cause of meningitis in HIV-infected patients with advanced immunosuppression and is associated with high morbidity and mortality in resource-limited settings [1]. *C. neoformans* spreads hematogenously from the lungs to the central nervous system causing fungal meningitis. Ocular manifestations are frequently observed during cryptococcal meningitis in AIDS patients. Papilledema, visual loss, and cranial nerve palsy are the most frequent ocular findings [2]. Intraocular involvement, due to hematogenous dissemination or extension through the leptomeninges, is unusual and includes choroiditis and chorioretinitis [3].

We report two cases of cryptococcal intraocular involvement in two AIDS patients with concomitant cryptococcal meningitis. Fluorescein (FA) and indocyanine green (ICG) angiographies as well as optical coherence tomography (OCT) findings are discussed.

## 2. Case 1

A 25-year-old man from Sub-Saharan Africa presented at our ophthalmology department for itchy red right eye since 3 weeks. The patient was infected with HIV following vertical transmission and diagnosed with HIV infection at the age of 7. The nadir CD4 count was 67/mm^3^. He was diagnosed with cryptococcal meningitis 9 months before. He received a treatment with liposomal amphotericin B and flucytosine followed by fluconazole secondary prophylaxis. After two months, he suffered from a relapse after voluntarily stopping his prophylaxis and the same treatment was given.

At presentation day in our clinic, the patient had again voluntarily stopped both antiretroviral treatment (elvitegravir/cobicistat/emtricitabine/tenofovir) and fluconazole 2 months before.

**Figure 1:**
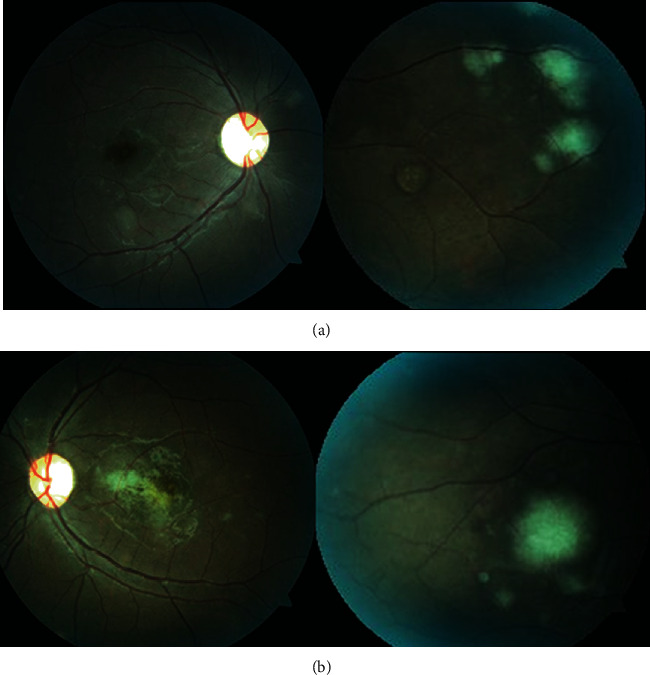
Fundus photograph of the (a) right and (b) left eyes displayed multiple yellowish subretinal lesions through the posterior pole and midperiphery in both eyes with a well-demarcated large deep macular lesion on the left eye.

**Figure 2:**
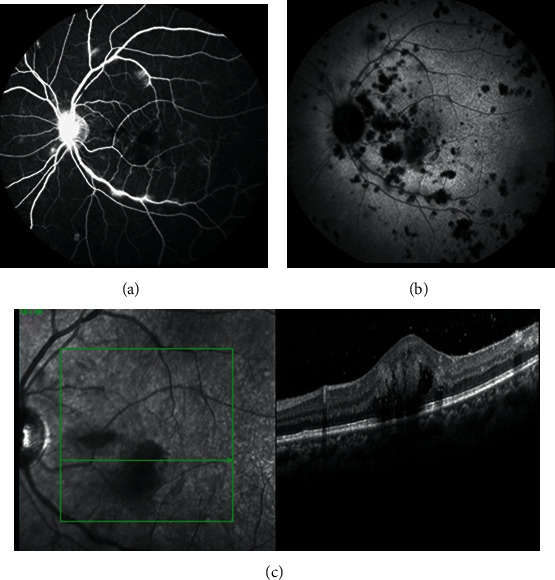
Case 1: left eye at presentation. (a) Late-phase fluorescein angiogram (FA) showed vasculitis of large venous vessels, hot disc, some macular hypofluorescent lesions, and mild diffusion at the level of some central lesion and midperipheral probably retinal lesions. (b) Indocyanine green angiography demonstrated numerous hypofluorescent spots, more numerous than the lesions seen on eye fundus and FA, suggesting associated widespread choroidal lesions. (c) Macular spectral domain optical coherence tomography demonstrated retinal infiltration in the inferior part of the macula with a diffuse thickening.

Visual acuity was 1/10 in the right eye and counting fingers in the left eye (LE); intraocular pressure was normal. Anterior chamber examination revealed bilateral conjunctival hyperemia, along with granulomatous keratic precipitates. Both eyes' fundus displayed mild vitritis along with multiple yellowish subretinal lesions through the posterior pole and midperiphery, with a well-demarcated large deep macular lesion on the LE (Figure 1). Late-phase fluorescein angiography (FA) of the LE showed vasculitis of large venous vessels, hot disc, some macular hypofluorescent lesions, and with a mild staining of some lesions (Figure 2(a)). ICG angiography demonstrated numerous hypocyanescent spots, more numerous than the lesions seen on eye fundus and FA, suggesting associated widespread choroidal lesions (Figure 2(b)). Macular spectral domain optical coherence tomography (SD-OCT) demonstrated retinal infiltration in the inferior part of the macula with a diffuse thickening (Figure 2(c)). Multimodal imaging findings were consistent with infectious bilateral panuveitis with multifocal chorioretinitis, vasculitis, and papillitis.

The patient was admitted, and laboratory investigations were performed. *C. neoformans* grew on multiples blood cultures with positive cryptococcal antigen (CRAG). A lumbar puncture demonstrated cerebrospinal fluid (CSF) lymphocytosis, hypoglycorrhachia, a positive CRAG, and *C. neoformans* culture. Vitreal culture was not performed. Computed tomography of the brain did not reveal any abnormalities, including signs of cerebral toxoplasmosis. Pulmonary tuberculosis was ruled out: both sputum and bronchoalveolar lavage direct examination, PCR, and culture were negative for *M. tuberculosis*. Blood culture was also negative for *Mycobacteria* sp. Physical examination was notable for papular verrucous skin lesions on the face. A skin biopsy was performed and PAS, Mucicarmine, and Grocott stainings revealed also the presence of *Cryptococcus*. Peripheral blood HIV viral load was 36400 copies/ml, and CD4+ T cells count was 61/mm^3^ (4% of total T cells).

A diagnosis of systemic and ocular cryptococcal infection was thus made, and combination therapy with liposomal amphotericin B (250 mg/24 h) and flucytosine (1750 mg/6 h) was restarted for a total of 14 days. The patient was discharged with fluconazole 800 mg along with antiretroviral therapy. At 10-month follow-up, peripheral blood HIV viral load was undetectable (<20 copies/ml) and CD4 count was 144/mm^3^. The patient was still taking fluconazole secondary prophylaxis. Visual acuity was, respectively, 10/10 and 4/10 and there was no sign of inflammation on the slit lamp. The eye fundus examination demonstrated multiple bilateral peripheral inactive lesions and a macular scar on the LE.

**Figure 3:**
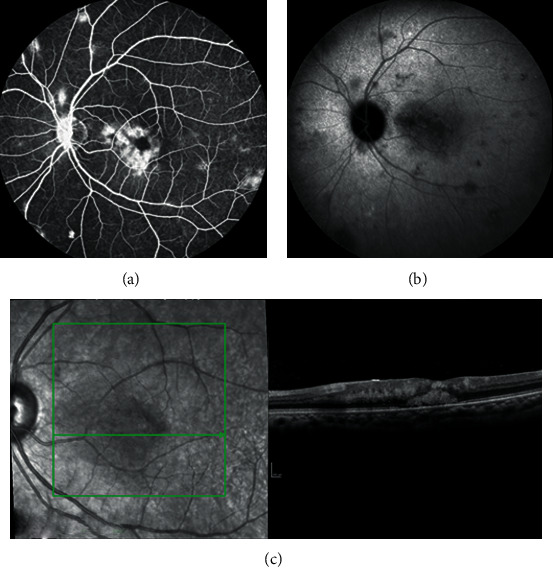
Case 1: left eye at 10-month follow-up. (a) Late-phase fluorescein angiogram showed regression of the vasculitis, remaining late diffusion of the optic nerve, and staining of the macular and peripheral scarring retinal lesions. (b) Late-phase ICGA demonstrated a drastic improvement with persistence of some discrete hypofluorescent spots probably corresponding to the scarring lesions seen on eye fundus and FA. (c) Spectral domain optical coherence tomography in the left eye showed a retrofoveolar scar with disruption of the retinal pigment epithelium and a loss of macular inner retinal structure.

Late-phase FA showed regression of the vasculitis, remaining late diffusion of the optic nerve, and staining of the macular and peripheral cicatricial retinal lesions (Figure 3(a)). Late-phase ICGA demonstrated a drastic improvement with persistence of some discrete hypocyanescent spots probably corresponding to the cicatricial lesions seen on eye fundus and FA (Figure 3(b)).

SD-OCT in the left eye showed a retrofoveolar scar with disruption of the retinal pigment epithelium and a loss of macular inner retinal structure (Figure 3(c)).

## 3. Case 2

**Figure 4:**
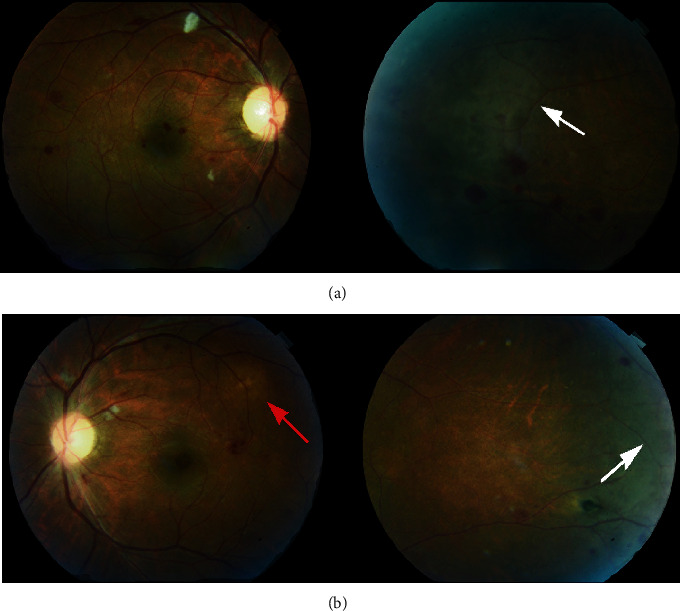
Case 2: fundus examination at presentation. Fundus photographs of the (a) right eye and the (b) left eye displaying multiple dot and blot hemorrhages, cotton wool spots, bilateral multiple deep yellowish lesions (red arrow), and large whitish peripheral retinal areas (white arrow).

**Figure 5:**
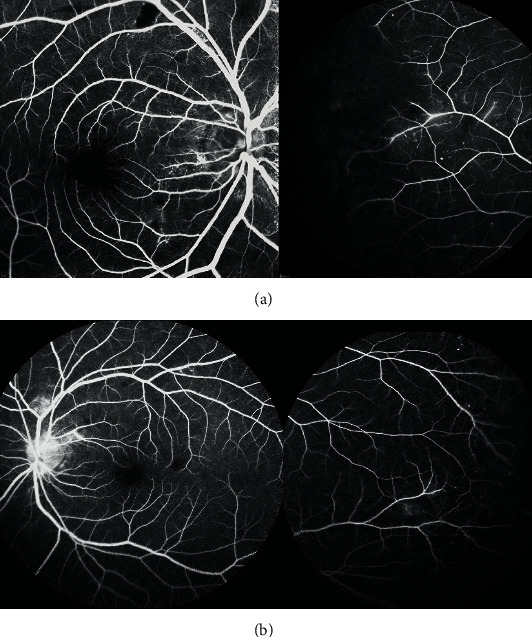
Case 2: fluorescein angiography findings at presentation. Asymmetric papillitis and occlusive vasculitis are seen in the (a) right and (b) left eyes.

**Figure 6:**
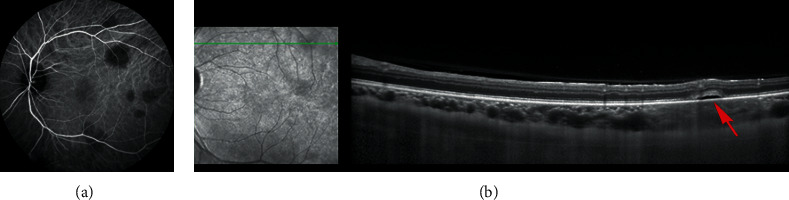
Case 2: ICG angiography and EDI-OCT at presentation (left eye). (a) Indocyanine green angiography demonstrated multiple hypofluorescent spots throughout the posterior pole and the midperiphery. (b) Enhanced Depth Imaging-OCT performed on a superotemporal hypocyanescent spot revealed a well-delineated lesion occupying all the choroidal space and considered as a cryptococcal granuloma, associated with a small serous retinal detachment (red arrow).

A 32-year-old man was admitted for confusion and loss of consciousness. HIV-1 testing was positive. CSF examination revealed positive CRAG and Indian ink examination. Both CSF culture and blood cultures grew for *C. neoformans*. Brain MRI did not reveal signs of cerebral toxoplasmosis. Pulmonary tuberculosis was ruled out: both sputum and bronchoalveolar lavage direct examination, PCR, and culture were negative for *M. tuberculosis*. Peripheral blood HIV-1 viral load was 800,000 copies/ml, and CD4+ T cell count was 12/mm^3^. On the third day of hospitalization, combination therapy by liposomal amphotericin B (350 mg/24 h) and flucytosine (100 mg/kg/24 h) was initiated and was stopped after 18 days because of acute kidney failure possibly related to liposomal amphotericin B and thrombocytopenia possibly related to flucytosine. Secondary prophylaxis by fluconazole 400 mg was initiated. Nevertheless, on the 30^th^ day of hospitalization, fever relapsed accompanied with headaches. Lumbar puncture analysis revealed positive Indian ink examination but negative *C. neoformans* culture. Consequently, liposomal amphotericin B treatment was reinitiated for probable relapse of cryptococcal meningitis. On the fifth day of hospitalization, the patient, complaining of floaters, was referred to the ophthalmology department. The visual acuity was 10/10 on both eyes. Slit lamp examination revealed few cells in the anterior chamber with no keratic precipitates or synechia. Fundus examination showed mild vitritis in both eyes associated to bilaterally multiple dot and blot hemorrhages, cotton wool spots, multiples deep yellowish lesions, and large whitish peripheral retinal areas (Figure 4). Vitreal culture was not performed. FA showed asymmetric papillitis and occlusive vasculitis in both eyes (Figure 5). ICGA demonstrated multiple hypocyanescent spots throughout the posterior pole and the midperiphery (Figure 6(a)). Enhanced Depth Imaging-OCT (EDI-OCT) performed on a superotemporal hypocyanescent spot in the left eye revealed a well-delineated lesion occupying all the choroidal space and considered as a cryptococcal granuloma, associated with a small serous retinal detachment which could be related to a secondary inflammatory response to the underlying choroidal lesion (Figure 6(b)).

All those findings were compatible with cryptococcal chorioretinitis associated with papillitis and occlusive vasculitis.

**Figure 7:**
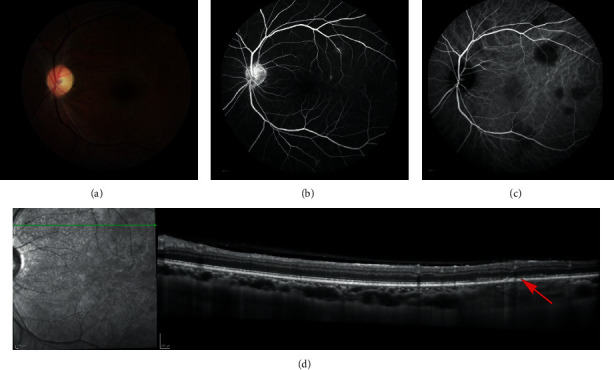
Case 2: left eye at 6-month follow-up. (a) Left eye fundus indicated a significant regression of the initial lesions with also resorption of hemorrhages and retinal cotton wools. (b) Late-phase FA showed decreased papillary diffusion with a remaining mild vasculitis on the posterior pole. (c) On ICGA, the hypocyanescent lesions are still present but decreased in size. (d) EDI-OCT showed resorption of the serous retinal detachment (red arrow).

At 6-month follow-up, LE fundus indicated a significant regression of the initial lesions with also resorption of hemorrhages and retinal cotton wools (Figure 7(a)). Late-phase FA showed decreased papillary diffusion with a remaining mild vasculitis on the posterior pole (Figure 7(b)). On ICGA, the hypocyanescent lesions were still present but decreased in size, suggestive of a healing process (Figure 7(c)). EDI-OCT showed resorption of the serous retinal detachment, a decrease in hyperreflectivity of choroidal infiltration and resorption of serous retinal detachment (Figure 7(d)).

## 4. Discussion

We report 2 rare cases of cryptococcal chorioretinitis associated with uveitis and vasculitis in 2 untreated AIDS patients with profound immunosuppression and concomitant cryptococcal meningitis.

Cryptococcal choroiditis represents an uncommon OI of persons with advanced HIV/AIDS. Visual loss in the setting of AIDS entails a broad differential diagnosis. Putative etiologies include HIV retinopathy, CMV retinitis, acute or progressive outer retinal necrosis (i.e., herpes simplex or varicella zoster virus-mediated), toxoplasma chorioretinitis, syphilitic chorioretinitis, *Pneumocystis jirovecii* choroiditis, ocular tuberculosis or Mycobacterium avium complex, candida, histoplasma or aspergillus endophthalmitis/vitritis, immune reconstitution uveitis, and lymphoma [4, 5]. Cryptococcal meningitis is frequently associated with ocular manifestations. In a study performed in Sub-Saharan Africa before the era of antiretroviral therapy on 80 patients with cryptococcal disease, papilledema was the most common ocular manifestation and was found in around 30% of the subjects [2]. Other frequent manifestations included cranial nerve palsy (9%) and loss of visual acuity (9%). Causes of bilateral visual loss included optic atrophy, optic neuropathy with abolishment of pupillary responses in one, and cortical blindness. Unilateral visual loss was caused by an occlusion of the central retinal vein with macular edema. Only two patients had signs of choroidal involvement: one with choroidal infiltrates and the other one with cryptococcoma. However, intraocular involvement following cryptococcal meningitis has rarely been reported [3–7]. The most common manifestation is choroiditis while anterior uveitis, vasculitis, and retinitis, as described in the present cases, are less common [6]. Previous reports indicate that the yeast can be directly isolated from intraocular tissues or fluids [6]. Dissemination likely occurs via the hematogenous route as illustrated in both cases with angiography exams that indicated both proximal and distal vessel lesions.

Interestingly, in both of our cases, chorioretinitis manifests during the course of treatment for cryptococcal meningitis. In the first case, the patient had already received months of treatment and chorioretinitis manifests in the context of a relapse. Similarly, in the second case, ocular inflammation occurs in the setting of a presumed relapse or clinical failure. Similar cases have been reported previously. Carney et al. reported cases of cryptococcal choroiditis in which choroiditis manifests 10 days after initiation of amphotericin B [7]. The authors proposed a scheme of progression of ocular infection following hematogenous dissemination: (1) cryptococcal choroiditis, (2) cryptococcal chorioretinitis, and (3) cryptococcal uveitis/vitritis/endophthalmitis [7].

Simultaneous involvement of all the different eye structures (anterior chamber, choroid, retina, and vessels) has rarely been reported in the course of disseminated *C. neoformans* infection, as well as reports describing multimodal imaging findings. Most cases were reported in patients with concomitant immunosuppression. Previous cases were reported in patients with advanced AIDS [8–10] or with T cell leukemia [11]. As described in the latter cases [8, 9], EDI-OCT and ICG have proven most useful in directing the diagnostic algorithm in panuveitis in immunosuppressed patients by defining the localization of lesions and assessing response to treatment. The two cases presented here highlight the potential of *C. neoformans* to infect all ocular structures including the optic nerve, retinal vessel, and choroidal stroma, leading, respectively, to papillitis, vasculitis, and choroiditis in immunocompromised patients. This involvement is found in some systemic granulomatous diseases, leading to the most frequent differential diagnoses of tuberculosis and sarcoidosis, but also syphilis, which remains the main masquerade syndrome in uveitis patients [12]. We excluded the possibility of intraocular cryptococcal/toxoplasma coinfection, because CT scan and MRI of the brain did not reveal signs of cerebral toxoplasmosis. Moreover, bilateral multifocal choroidal infiltrates are an uncommon presentation of ocular toxoplasmosis [13].

On the other hand, limited involvement of the eye presenting as uveitis with concomitant cryptococcal meningitis has been reported in nonimmunocompromised patients [14, 15].

In conclusion, these two cases reveal the potential of *C. neoformans* to infect all ocular structures mimicking other ocular diseases and this leads us to consider Cryptococcus as “a good masquerader” like syphilis and tuberculosis.
